# Consensus Review and Definition of Nonallergic Rhinitis With a Focus on Vasomotor Rhinitis, Proposed to be Known henceforth as Nonallergic Rhinopathy *Part 1. Introduction*

**DOI:** 10.1097/WOX.0b013e3181a8e146

**Published:** 2009-06-15

**Authors:** Michael A Kaliner, Judith R Farrar

**Affiliations:** 1 Institute for Asthma and Allergy, Chevy Chase, Md; 2 Life-Sciences Press, Washington, DC; 3 George Washington University School of Medicine, Washington, DC

**Keywords:** nonallergic vasomotor rhinitis, nonallergic rhinitis, vasomotor rhinitis, idiopathic rhinitis, nonallergic rhinopathy

## Abstract

"Nonallergic vasomotor rhinitis" (also referred to as nonallergic rhinitis and/or idiopathic rhinitis) is a term that has been used to describe a common nasal condition of unclear pathophysiology. Clinical options for patients are limited by a lack of straightforward diagnostic criteria and poorly defined and heterogeneous populations in clinical trials. A roundtable conference convened in December 2008 addressed these challenges. The outcomes were (1) a revised clinical definition and (2) appropriate inclusion and exclusion criteria (based on the revised definition) to be used for the enrollment of subjects in future clinical studies.

## 

The term nonallergic vasomotor rhinitis (also called idiopathic rhinitis) is generally agreed to describe rhinitis symptoms that occur in relation to nonallergic, noninfectious triggers such as (but not limited to) the following: changes in temperature, humidity, and barometric pressure; exposure to strong odors, tobacco smoke, and exhaust fumes; and even the ingestion of certain foods. Up to one third of patients with rhinitis are estimated to have nonallergic rhinitis, and close to 65% of patients with allergic rhinitis also have symptoms that occur or worsen in the presence of nonspecific, nonallergic stimuli.

The primary symptoms are nasal obstruction and rhinorrhea, with the term "vasomotor" suggesting involvement of neural, glandular, and vascular pathways. However, the term is misleading because it implies a known underlying mechanism when in reality the pathophysiology is not established. Additionally, the condition is associated with many other variable symptoms, and there is confusion about specific diagnostic testing and appropriate treatment options for these patients. The primary challenges associated with non-allergic vasomotor rhinitis are (1) agreement upon a definition that adequately describes this disease and (2) establishment of acceptable diagnostic criteria. An estimated 20 to 30 million Americans have this problem, and until criteria defining the disease are established, clinical studies examining effective treatment options are impossible to design.

In an attempt to address these challenges, a roundtable conference was convened in Washington, D.C. in December 2008 with the dual goals of (1) clinically defining nonallergic vasomotor rhinitis and then (2) using that definition to develop appropriate inclusion and exclusion criteria for the enrollment of subjects in future clinical studies.

Chaired by Michael A. Kaliner, MD, the consensus meeting included James N. Baraniuk, MD, Michael Benninger, MD, Jonathan A. Bernstein, MD, Phil Lieberman, MD, Eli O. Meltzer, MD, Robert M. Naclerio, MD, and Russell A. Settipane, MD. Each expert participant presented a state-of-the-art paper on a topic related to what is currently known about the pathophysiology, epidemiology, diagnostic criteria, and treatment options. The presentations were followed by discussion and counterpoints, leading to two consensus statements: one on the definition and the other on the study criteria. Our goal was to work toward improving our understanding of the phenotypes to identify homogeneous populations for studying the underlying mechanisms and developing better therapies. We have done that, and the findings, discussion, and recommendations are presented in these proceedings.

The NAR Consensus Panel Proceedings have been divided into 2 parts. Part 1, presented here, starts with an overview of the classification of nonallergic rhinitis syndromes and the proposal to change the terminology to reference these as "nonallergic rhinopathy"; it ends with a detailed consensus definition of the new term. However, this is presented with the caveat that the older terminology remains in the individual papers as they were written and submitted before the consensus discussion.

Part 2 includes papers on medications to treat nonallergic rhinopathy and concludes with a consensus discussion of the inclusion and exclusion criteria for clinical studies and remaining questions. This will be published in an upcoming issue of this journal.

## Note

The meeting was sponsored by the TREAT Foundation (Washington, DC) and supported by an unrestricted educational grant from Meda Pharmaceuticals. The funding company did not have any input into the development of the meeting or the proceedings; the company was not represented at the roundtable meeting.

